# Impact of Zinc- or Copper-Doped Mesoporous Bioactive Glass Nanoparticles on the Osteogenic Differentiation and Matrix Formation of Mesenchymal Stromal Cells

**DOI:** 10.3390/ma14081864

**Published:** 2021-04-09

**Authors:** Fabian Westhauser, Simon Decker, Qaisar Nawaz, Felix Rehder, Sebastian Wilkesmann, Arash Moghaddam, Elke Kunisch, Aldo R. Boccaccini

**Affiliations:** 1 Center of Orthopedics, Traumatology, and Spinal Cord Injury, Heidelberg University Hospital, Schlierbacher Landstraße 200a, 69118 Heidelberg, Germany; simon.decker@med.uni-heidelberg.de (S.D.); felix.rehder@med.uni-heidelberg.de (F.R.); sebastian.wilkesmann@med.uni-heidelberg.de (S.W.); elke.kunisch@med.uni-heidelberg.de (E.K.); 2 Institute of Biomaterials, University of Erlangen-Nuremberg, Cauerstraße 6, 91058 Erlangen, Germany; qaisar.nawaz@fau.de; 3 Center for Trauma Surgery, Orthopedics and Sports Medicine, ATORG—Aschaffenburg Trauma and Orthopedic Research Group, Klinikum Aschaffenburg-Alzenau, Am Hasenkopf 1, 63739 Aschaffenburg, Germany; Arash.Moghaddam-Alvandi@klinikum-ab-alz.de

**Keywords:** mesoporous bioactive glass nanoparticles, zinc, copper, osteogenic differentiation, extracellular matrix

## Abstract

Mesoporous bioactive glass nanoparticles (MBGNs) have gained relevance in bone tissue engineering, especially since they can be used as vectors for therapeutically active ions like zinc (Zn) or copper (Cu). In this study, the osteogenic properties of the ionic dissolution products (IDPs) of undoped MBGNs (composition in mol%: 70 SiO_2_, 30 CaO) and MBGNs doped with 5 mol% of either Zn (5Zn-MBGNs) or Cu (5Cu-MBGNs; compositions in mol%: 70 SiO_2_, 25 CaO, 5 ZnO/CuO) on human bone marrow-derived mesenchymal stromal cells were evaluated. Extracellular matrix (ECM) formation and calcification were assessed, as well as the IDPs’ influence on viability, cellular osteogenic differentiation and the expression of genes encoding for relevant members of the ECM. The IDPs of undoped MBGNs and 5Zn-MBGNs had a comparable influence on cell viability, while it was enhanced by IDPs of 5Cu-MBGNs compared to the other MBGNs. IDPs of 5Cu-MBGNs had slightly positive effects on ECM formation and calcification. 5Zn-MBGNs provided the most favorable pro-osteogenic properties since they increased not only cellular osteogenic differentiation and ECM-related gene expression but also ECM formation and calcification significantly. Future studies should analyze other relevant properties of MBGNs, such as their impact on angiogenesis.

## 1. Introduction

Mesoporous bioactive glasses have gained relevance in bone tissue engineering (BTE) applications, especially since they can be used as vectors for therapeutically active agents (ions, growth factors, etc.) that enhance or modify their intrinsic pro-osteogenic properties [1,2,3]. Among various others, possible candidate ions are zinc (Zn) and copper (Cu) since they either influence the bone metabolism itself, or they change other relevant features of biomaterials, such as their angiogenic or antibacterial properties [4,5,6,7]. For example, Zn promotes protein synthesis in osteoblasts and enhances the formation and mineralization of the osseous extracellular matrix (ECM) and its protein members [4,8,9,10]. The addition of Cu on implant coatings has been shown to stimulate not only fracture healing in a septic fracture model in rabbits but also prevented the formation of a biofilm on the respective titanium implants [7]. Furthermore, Cu has pro-angiogenic properties and also influences cellular osteogenic differentiation as well as ECM mineralization in a positive manner [5,6,11,12].

Due to their known beneficial properties, our group recently analyzed the osteogenic properties of sol–gel-derived mesoporous bioactive glass nanoparticles (MBGNs) with a composition of 70 mol% SiO_2_ and 30 mol% CaO doped with, inter alia, Zn or Cu when cultured in direct contact to human bone marrow-derived mesenchymal stromal cells (BMSCs) [13]. The mentioned study focused on the effects of the MBGNs on a cellular level. Thus the cellular osteogenic differentiation of the BMSCs was analyzed with the BMSCs being cultivated in direct contact with the MBGNs. A stimulating effect, especially of the expression profiles of genes encoding for members of the osteogenic ECM during the later stages of cultivation, was found. So far, the actual impact of the mentioned MBGNs on the ECM formation ability of MBGNs has not been analyzed, although ECM formation and maturation (i.e., calcification) play pivotal roles in bone regeneration [14,15]. The impact of biomaterials, such as MBGNs, on ECM formation and maturation, can be analyzed in cell culture settings using indirect cultivation approaches in which cells are cultivated in the presence of their ionic dissolution products (IDPs). These studies are necessary since direct cultivation settings complicate the assessment of the ECM, especially due to interactions with commonly used assay methods [16,17,18] caused by the intrinsic ability of bioactive glasses (BGs) to develop a carbonate-substituted hydroxyapatite layer on their surface and because BGs stick to their surroundings, also to the primitive ECM, in in vitro settings [2,16,19,20]. When transferred to the in vivo situation, indirect settings simulate the IDPs’ impact on the surrounding cells that are not in direct contact with the biomaterial itself [21]. These surrounding but not directly adjacent cells commit to matrix formation to a relevant extent making analyzing the IDPs’ impact in indirect in vitro settings relevant [22].

Therefore, in this follow-up study, the impact of the IDPs of undoped MBGNs (composition in mol%: 70 SiO_2_, 30 CaO) and MBGNs doped with 5 mol% of either Zn (5Zn-MBGNs) or Cu (5Cu-MBGNs) on the ECM formation and maturation ability of human BMSCs was evaluated using an indirect cultivation approach. The compositions studied were thus (in mol%): 70 SiO_2_, 25 CaO, 5 ZnO/CuO. Furthermore, the impact of the IDPs on viability and cellular osteogenic differentiation as well as on the expression of genes encoding for other relevant members of the ECM was assessed. Thus, the impact of MBGNs on those cells that commit to ECM production in the in vivo situation that are not directly adjacent to the material and are only exposed to the IDPs of MBGNs was assessed.

## 2. Materials and Methods

### 2.1. BG Production and Characterization

The preparation and characterization of MBGNs were conducted following protocols shown in our previous work [13]. In short, MBGNs were prepared using a modified Stöber method. MBGNs of 3 different compositions, i.e., undoped MBGNs (composition in mol%: 70 SiO_2_, 30 CaO) and Zn/Cu-doped MBGNs (composition in mol%: 70 SiO_2_, 25 CaO, 5 ZnO/CuO) were synthesized. After dissolving CTAB (cetyltrimethylammonium bromide; Merck, Darmstadt, Germany) in distilled water under continuous stirring for 15 min, ethyl acetate (VWR, Fontenay-sous-Bois, France) was added dropwise, while the pH was maintained at 10.2–10.4. Tetraethyl orthosilicate and calcium nitrate (both Sigma-Aldrich, Steinheim, Germany) were added to the solution, followed by the addition of corresponding metal salts, such as zinc nitrate hexahydrate or copper nitrate (both Sigma-Aldrich). After allowing the suspension to react for 4 h, centrifugation at 7830 rpm (Centrifuge 5430R; Eppendorf, Hamburg, Germany) was done to separate particles. The particles were dried in a furnace at 60 °C. The next day, the obtained particles were calcinated at 700 °C for 3 h.

Since the aim of the current work is to investigate the impact of IDPs of Zn- or Cu-doped MBGNs on the osteogenic differentiation and ECM formation of BMSCs, the further characterization of the synthesized MBGNs and their ion release kinetics at 1 mg MBGNs powder/mL of Dulbecco’s modified Eagle’s medium (DMEM) is not discussed here. Both aspects have already been discussed in our previous work [13].

### 2.2. Cell Origin, Ethics Approval

BMSCs of a 20-year-old male patient undergoing elective hip replacement surgery due to severe congenital hip dysplasia at the Heidelberg Orthopedic University Hospital were harvested. The patient’s written informed consent was obtained prior to cell collection. The study was approved by the responsible ethics committee of the Medical Faculty of the University of Heidelberg (S-340/2018).

### 2.3. BMSC Isolation and Cultivation

The isolation of BMSCs was performed following established protocols [23,24,25]. In short, mononuclear cells from donor bone marrow were extracted using a density gradient centrifugation protocol and seeded in 0.1% gelatin (Sigma-Aldrich)-coated T75 cell culture flasks (Sarstedt, Nümbrecht, Germany). Cells were cultivated in expansion medium (EM), consisting of DMEM high glucose, 12.5% *v/v* fetal calf serum (FCS), 2 mM L-glutamine, 1% *v/v* non-essential amino acids (NEAA), 50 µM β-mercaptoethanol (all Life Technologies, Darmstadt, Germany), 100 µg/mL penicillin/streptomycin (Sigma-Aldrich) and 4 ng/mL fibroblast growth factor 2 (Abcam, Cambridge, UK) at 37 °C and 5% CO_2_ in a humidified (≥95%) atmosphere. A first medium exchange was conducted after 24 h to wash off non-adherent cells. Thereafter, medium exchanges were performed twice weekly. Cells were passaged at 80% confluency and transferred to liquid nitrogen storage until further use. The experiments were conducted with BMSCs in passage 4.

### 2.4. Overview of the Study’s Experimental Design

To assess the influence of IDPs of the MBGNs on BMSCs, an indirect cultivation setting was used, as published previously [18]. The different MBGNs were added at a concentration of 0.5 mg/mL to cell culture medium (CCM; DMEM high glucose, 10% *v/v* FCS, 100 µg/mL penicillin/streptomycin) and incubated under cell culture conditions. Three days later, medium conditioned with the MBGNs’ IDPs was collected. The removed CCM volume was replaced by the same volume of fresh CCM. BMSCs were seeded in filtered IDP-containing CCM at a density of 18,400 cells/cm^2^ in 24- or 96-well plates (both Sarstedt), depending on the type of assay. For all assays, a control group with BMSCs seeded in regular CCM, without contact with the IDPs, was used. A medium exchange was performed twice a week. After 14 (D14) and 21 (D21) days, the different assessment methods were conducted.

### 2.5. Combined Assay for the Analysis of Cell Viability and Alkaline Phosphatase (ALP) Activity

Cell viability and alkaline phosphatase (ALP) activity as a marker of cellular osteogenic differentiation were assessed using a combined fluorescence-based assay following a previously published protocol [26]. Since it correlates with cell number and viability [27,28,29], metabolization of fluorescein diacetate (FDA) was quantified to determine cell viability. The conversion of 4-methylumbelliferyl phosphate (4-MUP), an ALP substrate, was measured as it correlates directly with ALP activity [26]. Following removal of CCM, cells were washed with Dulbecco’s phosphate-buffered saline (DPBS, Life Technologies; pH 7.0–7.3), then FDA substrate solution (0.1 mg/mL FDA (Sigma-Aldrich) in acetone (Carl Roth, Karlsruhe, Germany) 1:50 diluted in DPBS) was added. After staining at 37 °C for 5 min, cells were washed with DPBS before being lyzed with 0.5% Triton-X-100 (Sigma-Aldrich) at 37 °C for 5 min. Aliquots of the cell lysates were transferred to a white 96-well-plate (Kisker Biotech, Steinfurt, Germany) before addition of 4-MUP substrate solution (100 µM 4-MUP (Life Technologies) in ALP assay buffer composed of 75 mM TRIS pH 9.3, 1.5 mM MgCl_2_ and 0.15 mM ZnCl_2_ (all Carl Roth)) and incubation at 37 °C for 15 min. A fluorescence microplate reader (Wallac 1420 Victor 2; PerkinElmer, Waltham, MA, USA) was used to determine the emerging fluorescence at 485/530 nm (ex/em) for FDA and at 360/440 nm (ex/em) for 4-MUP. FDA fluorescence intensity was used as the basis for normalization of ALP activity.

### 2.6. Qualitative Analysis of Cell Morphology and Viability

A fluorescence microscopy-based live/dead-assay was performed to visualize cell morphology and viability. While FDA was applied for detection of viable cells, potentially remaining dead cells were visualized with propidium iodide (PI), as PI cannot pass viable cell membranes and, therefore, intercalates into DNA of compromised cells [27,28,29]. After discarding CCM, staining solution composed of 8 µg/mL FDA and 20 µg/mL PI (Life Technologies) in DPBS was added and incubated at 37 °C for 5 min. Following the disposal of the staining solution, cells were kept in DPBS and visualized using an Olympus IX-81 inverted fluorescence microscope (Olympus, Hamburg, Germany). Green (FDA) and red (PI) pictures were merged with ImageJ software (U.S. National Institutes of Health, Bethesda, MD, USA).

### 2.7. Gene Expression Analysis by qPCR

Assessment of osteogenic differentiation on a genetic level was conducted by a gene expression analysis of genes encoding for three important osseous ECM members, namely osteopontin (OPN), osteocalcin (OCN) and type I collagen alpha 1 (COL1A1) via quantitative real-time PCR (qPCR). After performing RNA isolation utilizing the PureLink RNA mini kit (Life Technologies) following the manufacturer’s instructions, 100 ng of RNA were reversely transcribed into cDNA, using a high-capacity RNA-to-cDNA kit (Life Technologies) following the manufacturer’s protocol. PowerUp SYBR Green master mix (Life Technologies) and the primer pairs shown in Table 1 were used to perform qPCR in a LineGene 9600 fluorescent quantitative detection system (Hangzhou Bioer Technology, Hangzhou, China). The expression of the analyzed genes was calculated with the ΔΔCt method by referring the target genes to the endogenous reference gene tyrosine 3-monooxygenase/tryptophan 5-monooxygenase activation protein zeta (YWHAZ) followed by normalizing the expression to the control group.

### 2.8. Quantification of ECM Collagen by Sirius Red Staining

Sirius red was used to quantify ECM collagen deposition because of its highly specific binding to collagen [18]. CCM was discarded, and the cells were washed with DPBS before fixation with 4% paraformaldehyde (PFA; Merck) in DPBS at room temperature for 60 min. Following fixation, three washing steps with distilled water were performed, then Sirius red staining solution, consisting of 1 mg/mL Sirius red F3BA (Chroma-Gesellschaft, Münster, Germany) in 1.3% picric acid (Sigma Diagnostics Inc., Livonia, MI, USA) in distilled water was added. After staining cells on a plate shaker at room temperature for 60 min, three washing steps with 0.01 M HCl (Carl Roth) followed. Bound dye was eluted by adding 0.1 M NaOH (Carl Roth) and incubating for 30 min on a plate shaker at room temperature. Aliquots of the eluted stain were transferred to a transparent 96-well plate (Sarstedt). A dilution series with known concentrations of Sirius red was added to the microtiter plate to determine the amount of eluted dye with a standard curve. Optical density at 570 nm was measured in a microplate reader (Autobio PHOmo; Autobio Diagnostics, Zhengzhou, China).

### 2.9. Quantification of ECM Calcification by Alizarin Red S Staining

Since it detects calcium deposits [25,30,31], staining with alizarin red S was utilized to determine ECM calcification. After removing CCM and washing cells with DPBS, cells were fixed with 70% ethanol (Serva Electrophoresis, Heidelberg, Germany) in distilled water at 6 °C for 20 min. Three washing steps with distilled water were performed before adding alizarin red S staining solution (0.5% alizarin red S (Waldeck, Münster, Germany) in distilled water). After staining for 10 min at room temperature, cells were washed four times with distilled water, and 10% cetylpyridinium chloride (Sigma-Aldrich) solution containing 10 mM sodium dihydrogen phosphate (AppliChem, Darmstadt, Germany) in distilled water was added to dissolve the bound dye. After a 5 min incubation on a plate shaker, aliquots of the dissolved stain were transferred to a transparent 96-well-plate (Sarstedt). A dilution series with known concentrations of alizarin red S was added to the microtiter plate to determine the amount of eluted dye with a standard curve. Optical density at 570 nm was measured in a microplate reader (Autobio PHOmo; Autobio Diagnostics).

### 2.10. Statistics

IBM SPSS Statistics (Version 25; IBM, Armonk, NY, USA) was used to conduct statistical analyses. Values were compared using Kruskal–Wallis and Mann–Whitney U tests with *p* < 0.05 as the level of significance. GraphPad Prism (Version 8.1.0; GraphPad Software, La Jolla, CA, USA) was used to design the graphs. Values are shown as rounded means with standard deviation where applicable. The number of biological replicates used for each measurement is *n* = 5. Except for the combined viability and ALP activity assay, measurements were performed in technical duplicates.

## 3. Results

### 3.1. The Presence of IDPs of MBGNs Decreased Cell Viability

**Figure 1:**
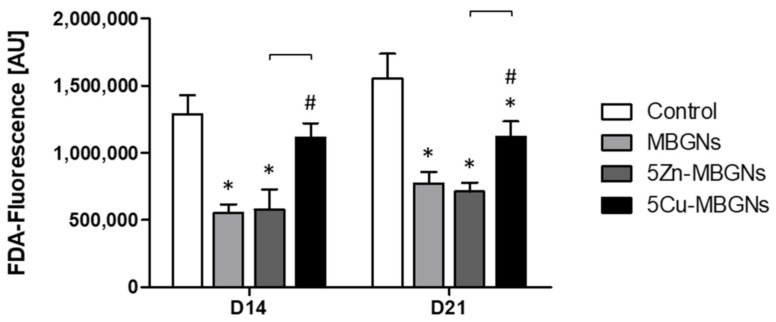
Viability of human bonemarrow-derived mesenchymal stromal cells (BMSCs). Significant differences compared to the control group are marked with (*), significant differences compared to mesoporous bioactive glass nanoparticles (MBGNs) with (#). Significant differences between the ion-doped MBGNs are highlighted with brackets.

Compared to the control group, the IDPs of MBGNs decreased cell viability and proliferation significantly (Figure 1). While 5Cu-MBGNs showed significantly higher viability than the other MBGNs, IDPs of the undoped MBGNs and the Zn-doped MBGNs had a comparable influence on the BMSC viability.

### 3.2. Influence of MBGNs’ IDPs on Cell Morphology and Viability

**Figure 2:**
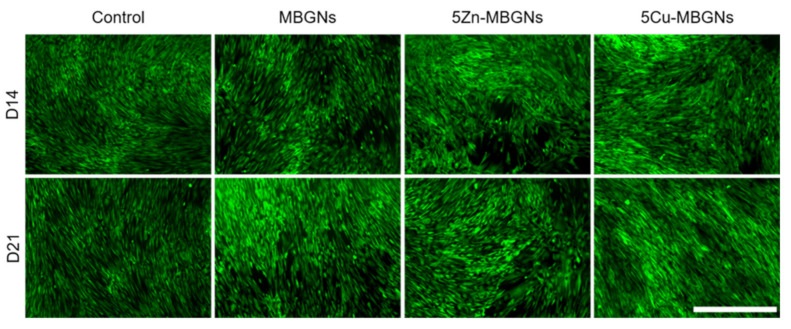
Representative live/dead-assay for the MBGN groups and the control group after an incubation time of 14 (D14) and 21 (D21) days. Viable cells are shown in green, compromised cells in red. Scale bar (bottom right corner) refers to 1000 μm and applies to all images. Magnification: 40-fold.

The number of green-stained cells in the MBGNs- and 5Zn-MBGNs groups on both days was approximately the same but appeared to be slightly lower compared to the control group (Figure 2). No remarkable difference regarding viable cells and cell density between the 5Cu-MBGNs and control group could be seen at both assessed time points. As cells already reached confluency in all groups after 14 days, no pronounced increase in density could be observed after 21 days. In general, only very few red-stained compromised cells were detectable, as dead detached cells were unintentionally removed with CCM.

### 3.3. IDPs of Undoped and Zn-Doped MBGNs Significantly Increased ALP Activity

**Figure 3:**
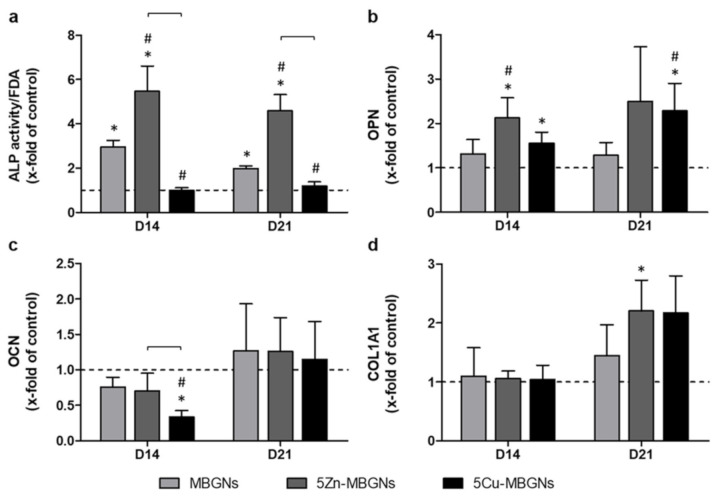
Alkaline phosphatase (ALP) activity of BMSCs (x-fold of control, normalized to fluorescein diacetate (FDA)) (**a**) and expression of genes encoding for relevant members of the extracellular matrix (ECM), namely osteopontin (OPN) (**b**), osteocalcin (OCN) (**c**) and type I collagen alpha 1 (COL1A1) (**d**). Values are normalized to the control group (indicated by the dashed line at y = 1). Significant differences compared to the control group are marked with (*), significant differences compared to MBGNs with (#). Significant differences between the ion-doped MBGNs are highlighted with brackets.

IDPs of 5Zn-MBGNs had the most pronounced positive effect on ALP activity, as activity levels were significantly higher compared to the untreated control group and all other MBGNs groups on D14 and D21 (Figure 3a). IDPs of undoped MBGNs also led to a significantly higher level of ALP activity compared to the control group as well as to the 5Cu-MBGNs group. ALP activity was approximately the same in the presence of IDPs of 5Cu-MBGNs when compared to the control group on both D14 and D21.

### 3.4. Influence of IDPs of MBGNs on ECM-Linked Gene Expression

On D14, expression of OPN was increased in all MBGNs groups when compared to the control group, with the 5Zn-MBGNs group being the only group to show significantly higher expression levels than the control group and the undoped MBGNs group (Figure 3b). Compared to the control group, IDPs of 5Cu-MBGNs significantly upregulated the OPN gene expression as well. Relative OCN expression on D14 was lower in the MBGNs groups compared to the control group (Figure 3c). 5Cu-MBGNs decreased OCN expression even to a significant extent when compared to the control group. OCN expression increased non-significantly in all MBGNs groups above the level of the control on D21. On D14, expression levels of COL1A1 were comparable for all groups (Figure 3d). On D21, COL1A1 expression increased in the presence of all MBGNs when compared to the control. In particular, IDPs of 5Zn-MBGNs and 5Cu-MBGNs led to COL1A1 expression levels twice as high as in the control group.

### 3.5. Zn-Doping of MBGNs Increases Collagenous ECM Formation and Calcification

**Figure 4:**
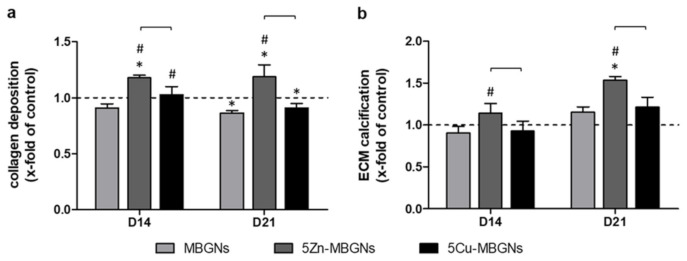
Collagen deposition (**a**) and ECM calcification (**b**) as markers for ECM formation and maturation. Values are normalized to the control group (indicated by the dashed line at y = 1). Significant differences compared to the control group are marked with (*), significant differences compared to MBGNs with (#). Significant differences between the ion-doped MBGNs are highlighted with brackets.

Collagen deposition in the group treated with IDPs of undoped MBGNs was slightly lower than in the control group after 14 days (Figure 4a). IDPs of 5Cu-MBGNs significantly increased collagen deposition compared to the undoped MBGNs group, but only a slight elevation compared to the control group could be seen after 14 days. 5Zn-MBGNs led to a significantly increased deposition of collagen compared to all other groups. On D21, collagen deposition in the 5Zn-MBGNs group stayed superior compared to all other groups, while IDPs of 5Cu-MBGNs and undoped MBGNs led to significantly lower collagen deposition compared to the control group.

On D14, IDPs of the undoped MBGNs and 5Cu-MBGNs had only limited effects on ECM calcification, while IDPs of 5Zn-MBGNs significantly increased calcification (Figure 4b). On D21, an increase in ECM calcification in all MBGNs groups was observable. ECM calcification in groups treated with IDPs of undoped MBGNs and 5Cu-MBGNs was non-significantly higher than the control group on D21. 5Zn-MBGNs induced significantly higher ECM calcification than any other group.

## 4. Discussion

Previously, our group assessed the impact of MBGNs based on the SiO_2_–CaO system on the osteogenic differentiation of BMSCs in a direct cultivation setting [13,32] in which cells are placed in direct contact with the MBGNs [16,17]. Using this approach, the influence of the direct physical contact of the cells to the MBGNs can be evaluated. Transferred to the physiological situation, the direct setting simulates the direct contact that exists when the material is used to fill a bone defect [21]. However, the IDPs of BGs are also transported by fluids to the surrounding tissue, represented by the indirect cultivation setting where cells are cultivated in media containing IDPs of the respective glasses, but not in their direct (physical) presence [16,17,18,21]. These surrounding but not directly adjacent cells play an important role in the formation and maturation of the ECM, making analyzing the impact of IDPs in indirect in vitro settings relevant [22]. Besides the differences in the transferability to the physiological situation, indirect cultivation settings also allow assessing the impact of BGs on the maturation and formation of the ECM without the effect of particle size or surface topography [13,18]. Furthermore, direct co-cultivation settings complicate the ECM assessment mostly due to the intrinsic ability of BGs to develop a carbonate-substituted hydroxyapatite layer on their surface upon contact with physiological liquids and because BGs stick to their surroundings, also to the primitive ECM in in vitro settings [2,16,19,20]. Therefore, to assess solely the impact of the IDPs of MBGNs alone and MBGNs doped with Zn or Cu on the ECM formation and maturation ability of BMSCs, an indirect cultivation setting was used, simulating the constant ion release of the MBGNs by parallel incubation as described previously [18].

### 4.1. About the Experimental Setup

The in vitro setting used in this study allows to monitor different phases during osteogenic differentiation of osteoblast precursor cells. For the interpretation of the results, it is important to outline the temporal sequence of osteogenic differentiation, which can be divided into three phases within the used experimental setting [14]. During the first phase ranging from day 1 to 4 in culture, cell numbers increase. From day 5 to 14, being the second phase of osteogenic differentiation, the cells shift from proliferation towards osteogenic differentiation characterized by the production of ALP. The activity of ALP acts as an osteoblastic marker enzyme in the setting used and is expected to peak between day 10 and 14 and represents the cellular differentiation from osteoblast precursor cells towards actual osteoblasts [14,28]. Starting after day 14 for a period of 2 weeks, the differentiated cells commit to the formation and maturation (i.e., calcification) of a primitive osseous ECM being the third phase of osteogenic differentiation in the used in vitro setting [14]. Since analyzing the latter was the major interest of this study, days 14 and 21 were chosen as evaluation time points to cover the relevant steps of ECM formation and maturation as well as the late phase of ALP production in order to prove that cellular differentiation resulted in the presence of osteoblastic cells. The impact of the MBGNs’ IDPs on ECM formation was evaluated by the analysis of the expression of genes encoding for relevant members of the ECM. As such, OPN, OCN and COL1A1 were chosen since they account for a relevant amount of ECM protein members [33]. Since collagen marks a major part of the osseous ECM, its deposition is an important marker of osteogenic commitment of BMSCs [27,34]. The deposition of collagen was quantified using the well-established Sirius red staining method [18]. ECM calcification takes place during maturation, being another part of the late stages of osteogenic differentiation, which was quantified by alizarin red staining [14]. The cells’ viability at the observation time points was also assessed, serving as an internal control.

### 4.2. Impact of Zn-MBGNs on Cell Viability

Compared to IDPs of the undoped MBGNs, IDPs of Zn-doped MBGNs did not have a relevant influence on BMSC viability, while the viability was significantly decreased compared to the control. Similar findings were described previously for different Zn-doped BGs [13,35,36]. The addition of Zn to the MBGNs, however, does not seem to diminish their biocompatibility. Also, cell growth patterns did not differ from cells cultivated in the presence of IDPs of undoped MBGNs.

### 4.3. Influence of Zn-MBGNS on Osteogenic Differentiation and on ECM Formation and Maturation

ALP activity was significantly upregulated by IDPs of Zn-doped MBGNs not only compared to the control but also in comparison to all other MBGNs groups. Oh et al. published corresponding results when culturing rodent BMSCs with Zn-containing sol–gel derived SiO_2_-30CaO BGs after 14 and 21 days in culture [37]. Zn also plays a role in the regulation of ALP activity: In 1986, Yamaguchi and co-workers described that Zn induces ALP activity and also enhances DNA synthesis resulting in a stimulation of bone growth in tissue derived from rodent femora [38]. Furthermore, Zn is relevant to support the activity and function of ALP [4,10]. IDPs of 5Zn-MBGNs did also upregulate OPN and OCN gene expression compared to both the control and the IDPs of undoped MBGNs. Furthermore, 5Zn-MBGNs IDPs increased collagen production and ECM calcification significantly when compared to undoped MBGNs or the control. Zn deficiency causes downregulation of ECM mineralization triggered by reduced ALP activity in osteoblasts outlining the important role of Zn in ECM mineralization [10,39]. Huang and co-workers demonstrated that exposure of human dental pulp stem cells towards IDPs of zinc-containing BGs led to an increase of the presence of mineralized nodules [40]. Saino et al. analyzed the impact of Zn-doped 58S BGs on Saos-2 cells and found not only an increase in ALP activity, but also significantly higher calcium deposits in the groups cultivated in the presence of Zn-doped BGs [41]. Thus, 5Zn-MBGNs provide the most favorable pro-osteogenic properties in this setting, qualifying them as possible candidates for further application in BTE, for example, in in vivo models.

### 4.4. Impact of Cu-MBGNs on Cell Viability

IDPs of Cu-doped MBGNs had a positive influence on the viability of the BMSCs when compared to IDPs of the undoped MBGNs. However, compared to the control group, viability was also significantly decreased at D21. There are contradictory findings of the influence of Cu on the viability of BMSCs when cultured with Cu-containing BGs. In our previous study, cell viability was increased when BMSCs were cultured in the direct presence of 5Cu-MBGNs [13]. Wu and coworkers found a reduced cell number when BMSCs were cultured in the presence of IDPs of Cu-doped mesoporous BGs compared to their undoped controls [3].

### 4.5. Influence of Cu-MBGNs on Osteogenic Differentiation and on ECM Formation and Maturation

While Rodríguez and co-workers reported negative effects of Cu on the proliferation of BMSCs isolated from the bone marrow of postmenopausal women on one hand, on the other hand, the osteogenic differentiation of BMSCs was increased [5]. Furthermore, the presence of Cu also increased the ECM mineralization of BMSCs after 12 days of incubation while ALP activity was reduced [5]. Compared to IDPs of undoped MBGNs, ALP activity was also reduced in the presence of 5Cu-MBGNs’ IDPs and remained on the level of the control that was cultivated in regular CCM. Similar findings were reported by Li and co-workers analyzing the impact of CuSO_4_ on rodent BMSCs after seven days of cultivation: in the presence of CuSO_4_, ALP activity decreased as well as the expression of the OCN, OPN and COL1A1 genes [42]. However, in the present study, IDPs of 5Cu-MBGNs increased OPN and COL1A1 expression above the controls’ level and exceeded the expression levels of cells cultivated in the presence of IDPs of undoped MBGNs. Compared to the study of Li, not only different cells (rodent vs. human BMSCs) but also different evaluation time points were used: likely, the expression of OCN, OPN and COL1A1 as genes being regulated in the later phases of osteogenic differentiation is not yet fully regulated after seven days of culture [42]. Furthermore, care must be taken when comparing results of studies using different methodological settings: for example, BMSCs react differently towards exposure to the well-known 45S5-BG compared to other “osteoblast-like” cell lines as demonstrated previously [43]. On D14, IDPs of 5Cu-MBGNs increased collagen production resulting in increased ECM calcification at D21. However, the observed elevation of calcification was—in contrast to the data published by Rodríguez and co-workers—not significant [5]. Cu enhances the angiogenic properties of biomaterials: for example, Weng and co-workers showed that co-doping with Cu and strontium enhances both osteogenesis and angiogenesis of BG nanofibers [44]. Rath and co-workers investigated Cu-doped BG scaffolds in a co-culture model of BMSCs and human dermal microvascular endothelial cells (HDMECs). They found high vascular endothelial growth factor (VEGF) levels secreted by BMSCs, resulting in the formation of tubular structures by the HDMECs, demonstrating the relevant interaction of BMSCs and vascularization [45]. Since cells in cell culture settings are supplied with oxygen and nutrients by diffusion and not by vascularization, the actual impact on bone formation might not be reflected to its full extent in the used 2D cell culture setting: since bone regeneration is an energy-demanding process [46], vascularization needs to be optimized to support bone regeneration in the in vivo situation. For example, our group compared the boron-containing 0106-B1-BG to 45S5-BG in an in vitro setting using BMSCs and in vivo in an ectopic mouse model in a previously published study [47]. Like Cu, boron is also used to improve the angiogenic properties of BGs [20,48,49]. While the impact on osteogenic differentiation in vitro was comparable between the BG groups, the formation of osteoid in vivo was significantly higher in scaffolds made from 0106-B1-BG compared to scaffolds made from 45S5-BG, mediated by improved vascularization in the 0106-B1-BG group [47]. In summary, IDPs of Cu-doped MBGNs had slightly positive effects on the late stages of osteogenic differentiation and on ECM formation and calcification in this study. Considering the fact that Cu is mostly used as a pro-angiogenic ion in BGs [45,50,51], together with its slightly positive influence on osteogenic differentiation, it qualifies for the use as an additional dopant for BGs intended for the use in BTE.

## 5. Conclusions

The impact of MBGNs on the formation and maturation of the ECM is significantly influenced by the presence of ions with certain therapeutic activity. IDPs of the Zn-doped MBGNs had a comparable influence on the cell viability like IDPs of undoped MBGNs, while IDPs of 5Cu-MBGNs even enhanced cell viability compared to IDPs of undoped MBGNs and 5Zn-MBGNs. Cu-doped MBGNs’ IDPs had slightly positive effects on the late stages of osteogenic differentiation and on ECM formation and calcification. 5Zn-MBGNs provided the most favorable pro-osteogenic properties in this setting, since they increased not only ALP activity or the expression of OCN and COL1A1 but also ECM formation and calcification significantly. Due to the known pro-angiogenic properties of Cu, co-doping of MBGNs with Cu and Zn might enhance both the MBGNs’ pro-osteogenic and their pro-angiogenic properties. Therefore, future studies should focus on the evaluation of co-doped MBGNs.

## Figures and Tables

**Table 1:** qPCR primer pairs. tyrosine 3-monooxygenase/tryptophan 5-monooxygenase activation protein zeta (YWHAZ; reference gene), secreted phosphoprotein 1/osteopontin (SPP1/OPN), osteocalcin (OCN), type I collagen alpha 1 (COL1A1).

Gene	Forward (5′ → 3′)	Reverse (5′ → 3′)
YWHAZ	TGC TTG CAT CCC ACA GAC TA	AGG CAG ACA ATG ACA GAC CA
OPN	GCT AAA CCC TGA CCC ATC TC	ATA ACT GTC CTT CCC ACG GC
OCN	ACC GAG ACA CCA TGA GAG CC	GCT TGG ACA CAA AGG CTG CAC
COL1A1	GTG GCC TGC CTG GTG AG	GCA CCA TCA TTT CCA CGA GC

## Data Availability

All relevant data are shown within the manuscript. Further data are available on request from the corresponding authors.
